# Strong male-biased operational sex ratio in a breeding population of loggerhead turtles (*Caretta caretta*) inferred by paternal genotype reconstruction analysis

**DOI:** 10.1002/ece3.761

**Published:** 2013-10-30

**Authors:** Jacob A Lasala, J Scott Harrison, Kris L Williams, David C Rostal

**Affiliations:** 1 Department of Biology, Georgia Southern University, BIOSC 99994324 Old Register Road, Statesboro, Georgia, 30460; 2 Department of Biology, Florida Atlantic University777 Glades Road, Boca Raton, Florida, 33431; 3 Caretta Research ProjectP.O. Box 9841, Savannah, Georgia, 31412

**Keywords:** *Caretta caretta*, conservation genetics, microsatellites, Northwest Atlantic Ocean, paternal contributions, polyandry

## Abstract

Characterization of a species mating systems is fundamental for understanding the natural history and evolution of that species. Polyandry can result in the multiple paternity of progeny arrays. The only previous study of the loggerhead turtle (*Caretta caretta*) in the USA showed that within the large peninsular Florida subpopulation, multiple paternity occurs in approximately 30% of clutches. Our study tested clutches from the smaller northern subpopulation for the presence of multiple paternal contributions. We examined mothers and up to 20 offspring from 19.5% of clutches laid across three nesting seasons (2008–2010) on the small nesting beach on Wassaw Island, Georgia, USA. We found that 75% of clutches sampled had multiple fathers with an average of 2.65 fathers per nest (1–7 fathers found). The average number of fathers per clutch varied among years and increased with female size. There was no relationship between number of fathers and hatching success. Finally, we found 195 individual paternal genotypes and determined that each male contributed to no more than a single clutch over the 3-year sampling period. Together these results suggest that the operational sex ratio is male-biased at this site.

## Introduction

Characterization of a species mating system is an important part of understanding its natural history (Wright 1931; Bjorndal et al. 1983). Mating behavior, including mate choice (Bateson 1983), mate guarding (Grafen and Ridley 1983), social pair bonds (Cezilly et al. 2000), extra-pair copulations (Barash and Lipton 2001) as well as breeding adaptations such as number of females mating with males (Searcy and Yasukawa 1995) or number of males mating with females (Arnqvist and Nilsson 2000) are all important aspects of mating systems. Mating systems are especially important within small populations as they can affect the genetic effective population size (*N*_e_) and the evolution of that species (Wright 1931; Frankham 1995; Vucetich et al. 1997; Arden and Kapuscinski 2002; Charlesworth 2009).

A species mating system is often described by studying a single population and then drawing inferences to the whole of the species (Refsnider 2009; Beasley et al. 2010; Yue and Chang 2010). Variation among populations and gaps in our understanding of a species are often revealed when studies are compared over many locations (Dutton et al. 1999; Crim et al. 2002; Jensen et al. 2006; Bowen and Karl 2007). This is to be expected as populations within a species can experience different environmental and demographic variables that influence factors such as breeding, mate availability, mate quality, and mate competitiveness. Assessment of variation among populations is important in building a more complete definition of that species mating system.

Here, we discuss the mating system of the loggerhead marine turtle *(Caretta caretta*). Loggerheads, like other marine turtles, are long lived and typically reach sexual maturity between 20 and 35 years of age (Snover 2002; Conant et al. 2009; Turtle Expert Working Group 2009). Once sexually mature, male loggerheads are able to mate yearly, while adult females typically mate every 2–3 years (Wibbels et al. 1990; Owens 1997; Cason 2009; Conant et al. 2009). Observations of loggerheads in the coastal waters off Georgia and northeast Florida suggest mating occurs from late March through early June (Dodd 1988; Frick et al. 2000). Loggerhead courtship and mating have been observed to occur nearshore of adjacent rookery beaches and within the riverine sounds separating barrier island-type nesting beaches (Dodd 1988; Frick et al. 2000). Females can store sperm and mate with more than one male in a single season (Owens 1980; Pearse and Avise 2001; Moore and Ball 2002; Sakaoka et al. 2013). During mating events, both single mating pairs as well as single females with several satellite males have been observed (Caldwell et al. 1959; Frick et al. 2000). Several studies suggest that males exhibit aggressive behavior toward each other to gain access to a female (Caldwell et al. 1959; Schofield et al. 2006). After mating, males return to foraging grounds while females remain in the vicinity of the nesting beach (Limpus and Reed 1985; Dodd 1988). In the Northwest Atlantic, females travel to nesting beaches between late April and early September depositing 3–7 clutches (typically 5) with approximately 14 days between nesting events (Caldwell 1962; Miller 1997; Drake 2001; Conant et al. 2009).

Most of the behavioral knowledge we have of loggerhead mating systems comes from nesting beach-based studies. Systematic and direct observation of the mating behavior of marine turtles in the wild is difficult (Booth and Peters 1972; Limpus 1993). The numbers of mates are not typically quantified, and numbers of males mounting a female do not directly reflect the number of males contributing to a clutch (Owens 1997). As adult female marine turtles return to their natal regions to nest, the nesting aspects of their reproductive behavior are more accessible to direct observation (Bjorndal et al. 1983; Dodd 1988; Bowen et al. 2004). Adult male marine turtles, which typically remain in open waters, are less accessible to direct observation. The published studies of marine turtle mating behavior are limited in sample size and observation periods are often brief (Caldwell et al. 1959; Caldwell 1962; Frick et al. 2000). In addition, quantification of promiscuity, breeding locations, and the nature of mating behavior generally remains obscure (Caldwell et al. 1959; Dodd 1988; Frick et al. 2000; Schofield et al. 2006).

Population genetic studies of loggerhead turtles in the Northwest Atlantic show a strong signature of sex-biased migration and gene flow. Maternally inherited mitochondrial markers show high levels of variation among nesting beach populations indicating no gene flow among them (Bowen et al. 2005; Shamblin et al. 2011). In contrast, bi-parentally inherited microsatellite markers indicate high levels of gene flown between these same populations (Bowen et al. 2005). These contrasting results suggest that the population structure of the loggerhead in the Northwest Atlantic involves a dynamic interaction between male migration and mating and female nest site fidelity (Bowen et al. 2005; Shamblin et al. 2011). Loggerhead populations in Japan (the endangered North Pacific Ocean discrete population segment) also show patterns consistent with high levels of male-mediated gene flow by migration through courtship areas (Watanabe et al. 2011).

Our understanding of the loggerhead mating system is incomplete because most studies do not assess the number of males contributing to nesting populations. This is a relevant variable for the conservation and management of this species and the Northwest Atlantic Ocean loggerhead discrete population segment which is designated as threatened (Federal Register 2011). Few studies have quantified the number of males contributing to a loggerhead discrete population segment, subpopulation or nesting assemblage largely because counting males is difficult as they are not accessible on land (Moore and Ball 2002; Zbinden et al. 2007).

Using genetic techniques to examine the paternity of hatchlings, our study evaluated the number of sires for a nesting beach indirectly. By comparing male genotypes, we can estimate contributing males without having to witness the mating itself. Exclusion paternity analysis can estimate the potential genotypes of contributing fathers. This allows an estimation of the number of individuals contributing to nesting sites. Previous studies of paternity in marine turtles indicate that multiple paternity is common (Table 1). As hatchling sex ratios may become increasingly female-biased due to global climate change (Hanson et al. 1998; Delgado et al. 2010; LeBlanc et al. 2012; Wright et al. 2012a,b), it is important to determine how many males actually contribute to a nesting population so that baselines are established for future comparison.

**Table 1:** Previous studies of multiple paternity in sea turtles.

Species	# Clutches Analyzed	% Multiple Paternity	Citation
*Caretta caretta*	70	31 (22/70)	Moore and Ball (2002)
*Caretta caretta*	20	95 (19/20)	Zbinden et al. (2007)
*Caretta caretta*	7	43 (3/7)	Sakaoka et al. (2011)
*Caretta caretta*	11	27 (3/11)	Sakaoka et al. (2013)
*Chelonia mydas*	22	9 (2/22)	FitzSimmons et al. (1997)
*Chelonia mydas*	18	61 (11/18)	Lee and Hays (2004)
*Chelonia mydas*	20	30 (6/20)	Wright et al. (2012a,b)
*Dermochelys coriacea*	20	10 (2/20)	Crim et al. (2002)
*Dermochelys coriacea*	38	42 (5/12)	Stewart and Dutton (2011)
*Eretmochelys imbricata*	10	20 (2/10)	Joseph and Shaw (2011)
*Eretmochelys imbricata*	43	9 (4/43)	Phillips et al. (2013)
*Lepidochelys olivacea*	13	30 (4/13)	Jensen et al. (2006)
*Lepidochelys olivacea*	13	92 (12/13)	Jensen et al. (2006)
*Lepidochelys kempi*	26	58 (15/26)	Kichler et al. (1999)

The overarching goal of this study was to examine several facets of the mating system within the full nesting population of Wassaw Island, GA; a small nesting assemblage within the Northwestern Atlantic Ocean discrete population segment. The three objectives of this study are as follows: (1) to quantify the frequency of multiple paternity clutches within the full nesting population of the small rookery of Wassaw Island, Georgia, USA; (2) to estimate the number of males contributing to the Wassaw Island clutches; and (3) to determine if correlations exist between the number of fathers per clutch and female size, hatching success and/or the year sampled.

## Methods

### Field methods

Samples were obtained during three nesting seasons (2008–2010) on the 11.3 km beach of Wassaw National Wildlife Refuge (Wassaw Island, GA, USA). Wassaw Island is a peripheral small nesting population within the Northwest Atlantic Ocean discrete population segment. Nesting female samples were collected from May until early August, and hatchling samples were collected from mid-July until early October in 2008, 2009, 2010. The nesting seasons were partitioned into subseasons (Early: 5/15-6/10; Middle: 6/11-7/8; Late: 7/9-8/4) defined by maternal hormonal titers (Drake 2001) so that we could be sure the entire nesting season was represented.

Adult nesting females were identified and tagged during nightly patrols with the Caretta Research Project. Over the 3 years of sampling, 119 loggerhead nests were laid in 2008, 91 nests were laid in 2009, and 162 were laid in 2010. Each year, ten individual females from each subseason (Early, Middle and Late) were tagged and measured according to US Fish and Wildlife protocols (Barnard and Keinath 1999; Williams and Frick 2000). Blood samples were taken from the cervical sinus using a 21Gx1-1/2” needle and retained in a 5-mL sodium heparin vacutainer (Owens and Ruiz 1980). According to the sampling design, no individual would be sampled more than once. Over the three-season study period, 90 individual females were sampled.

Nests of sampled females were caged promptly to prevent predation as well as the escape of hatchlings. A GPS reading was taken to mark the location of each nest. Stakes were added at the dune line to provide redundant locators for nest from the water line and to document the date the clutch was laid. Over the course of the nesting season, some clutches were lost due to storms/flooding: seven clutches were lost in 2008, nine were lost in 2009, and two were lost in 2010. The remaining 72 clutches were analyzed: at hatching, up to 20 hatchlings were collected randomly from each loggerhead nest, weighed, measured, and euthanized. Residual yolk sacs were removed from these hatchlings, weighed and stored at −20°C. Once yolk sacs were removed for analysis, the rest of each hatchling was used for additional unrelated studies.

### Genotyping

Maternal DNA was extracted by adding 2 μL of blood to 50 μL lysis buffer (10 mM Tris pH 8.3, 50 mM KCl, 0.5% Tween 20, and 200 μg/mL proteinase k) and incubated at 65°C for 1 h followed by 100°C for 15 min. Hatchling DNA was extracted from residual yolk sacs using the DNeasy blood and tissue kit (Qiagen, Valencia, CA) following manufacturer protocol. Polymerase chain reaction (PCR) amplification was carried out using primers for five microsatellite loci (CcP7E05, CcP2F11, CcP7D04, CcP7C06, and CcP8D06) designed for *Caretta caretta* (Shamblin et al. 2009). PCRs were carried out as a multiplex reaction in 25-μL volumes, consisting of 2 μL of extracted DNA, 10 μL of Apex Taq Master Mix (Genesee Scientific, San Diego, CA), 0.8 μM CcP7E05 Forward and Reverse, 0.4 μM CcP2F11 F&R, 2 μM CcP7D04 F&R, 0.4 μM CcP7C06 F&R, 2 μM CcP8D06 F&R, and 8 μL dIH_2_0. Thermocycling protocol was as follows: 95°C for 5 min; 40 cycles of 95°C for 20 s; 60°C for 30 s and 72°C for 30 s; and 72°C for 10 min.

PCR products were analyzed using an ABI 3500 Genetic Analyzer. Alleles were sized at each locus in relation to an internal size standard using GeneMapper 3.0 software (Applied Biosystems, Foster City, CA). Microsatellite loci were checked for null alleles using Micro-Checker 2.2.3 (Van Oosterhout et al. 2004). Observed and expected heterozygosity and deviations from Hardy–Weinberg Equilibrium were calculated for the maternal samples and assessed using GenAlEx and GDA (Lewis and Zaykin 2000).

### Paternity analysis

Paternity was evaluated with paternity exclusion analyses. Each hatchling's individual multilocus genotype was determined and when known maternal alleles were subtracted from each locus, the remaining paternal alleles formed suspected paternal genotypes. This analysis was performed using the programs GERUD 2.0 and COLONY 2.0. GERUD 2.0 assesses the minimum number of fathers per clutch (Jones 2005; Zbinden et al. 2007; Jones et al. 2010; Yue and Chang 2010) and can be conservative in its estimates of the number of paternal contributions. COLONY 2.0 is a maximum likelihood-based program that determines the maximum number of fathers per clutch. GERUD 2.0 fails if there are more than 6 fathers, so COLONY 2.0 was used to repeat the analysis on clutches that GERUD 2.0 could not evaluate. Using both programs, we approximated a range, where GERUD 2.0 was the minimum and COLONY 2.0 was the maximum.

COLONY 2.0 was also used to compare sibling relatedness for all the clutches (Jones and Wang 2010). In this analysis, the error rate of genotyping was set to 0.025 as suggested by Wang (2004). The determination of multiple paternity within a clutch was established by the occurrence of more than two paternal alleles over at least two loci–this allowed for the possibility of a mutation at one locus (Yue and Chang 2010). To determine whether paternal contributions were significantly different from equality in each clutch, goodness-of-fit χ^2^-tests were run on all 3 years of data. When running χ^2^-tests, the years were separated as we assume that each year is independent of one another.

The probability of identity was estimated using the method employed by GenAlEx within our 3-year data set. The probability of identity provides an estimate of the average probability that two samples will have the exact same genotype given the estimated allele frequencies of the loci used. The probability of exclusion (when only one parent is known) was also determined using GenAlEx to estimate the statistical power of our individual loci and our combined loci.

Each paternal genotype was compared to determine whether any of the estimated paternal genotypes were sampled more than once using COLONY 2.0 and GenA1Ex. Further, using COLONY, we compared all sampled clutches to these genotypes to determine the number of clutches to which a predicted individual male contributed. Probability of identity values and probability of exclusion (proportion of the population that has a genotype composed of at least one allele not observed in the mixed profile) when only one parent is known were determined using GenAlEx 6.41 (Peakall and Smouse 2006).

### Statistical analysis

All 3 years were analyzed together (2008–2010). Every test that was performed using data from GERUD was also performed using data from COLONY. All analyses were carried out using the program SAS 9.3 (SAS Institute Inc., Cary, NC).

Our assumptions were that female characteristics vary randomly by year and nesting events by individual turtles are independent of one another. We sampled unique females (no repeat nesters). We fitted the data to a generalized linear model with a gamma distribution. As our data were left skewed, the gamma distribution was the best model to compare variability in number of fathers by to year, date and female size. We ran the model twice (once for each paternity program), with the number of fathers/clutch as the dependent variable. Female size (straight carapace length (cm)), the Julian date the clutches were laid, and the year the clutch was laid (2008, 2009, or 2010) were all defined as independent variables. We ran the initial model with all the variables and then we removed the independent variable with the highest *P*-value, to assess the relationship between the variables. This step by step removal of variables determined which variables had no effect on the number of fathers per clutch. We continued removing the highest *P*-value until the only independent variable that remained had *P*-value <0.05.

We ran a separate generalized linear model (with a gamma distribution) to determine whether the number of fathers per clutch affected hatching success of each clutch (percentage of hatchlings that did *not* emerge from each nest). Hatching success was defined as the dependent variable, and the number of fathers per clutch defined as the independent variable.

## Results

The five microsatellite markers amplified consistently in all samples. Combining all five loci produced an expected probability of exclusion of 99.64%, with the assumption that only one parental genotype would be known (Table 2). The combined probability of identity using the five loci was 1.5 × 10^−6^. Following the protocol of the program Pedant, it was determined that the combined allelic dropout rate of the samples was 0.0254, and the false allele rate was 0.1070 (Johnson and Haydon 2007). Allele number ranged from 12 for the locus CcP7C06 to 27 for the locus CcP8D06 (Table 2). Deviations from Hardy–Weinberg were not found among the maternal samples.

**Table 2:** Descriptive statistics of the five polymorphic microsatellite markers. Number of alleles (A), expected heterozygosity (H_E_), and observed heterozygosity (H_O_).

Locus	Size Range (bp)	Dye	A	H_E_	H_O_	Expected Exclusion Probability
CcP7E05	164–236	6FAM	18	0.920	0.978	0.695
CcP2F11	252–308	6FAM	16	0.892	0.956	0.626
CcP7D04	320–376	6FAM	14	0.907	0.913	0.669
CcP7C06	256–296	HEX	12	0.864	0.858	0.541
CcP8D06	256–376	TAMRA	27	0.941	0.956	0.792

Of the original 90 females' nests, 72 clutches survived: 23 from 2008, 21 from 2009, and 28 from 2010. Separating by nesting period, there were 23 early nests, 26 middle nests, and 23 late nests. Clutches were laid from May 25 to July 17. A total of 1,282 hatchlings were sampled and analyzed. The average number of hatchlings genotyped per clutch was 18.2 (Range = 5–20; SD = 4.3). Permit regulations limit sampling to 20% of a clutch or up to 20 hatchlings. This resulted in six nests with a sample size <10 hatchlings. Over the 3-year period, the average clutch size was 114.7 (Range = 52–168, SD = 23.9) and the average percentage of hatchlings sampled from a clutch was 16.5% (Range = 3.3–24.7, SD = 5.7). The average percentage of hatchlings that did not emerge from a clutch was 21.7% (Range = 4.9–67.2, SD = 13.7). Average female size (straight carapace length) over the 3 years was 98.6 cm (Range = 84.5–111.0, SD = 6.5).

**Figure 1:**
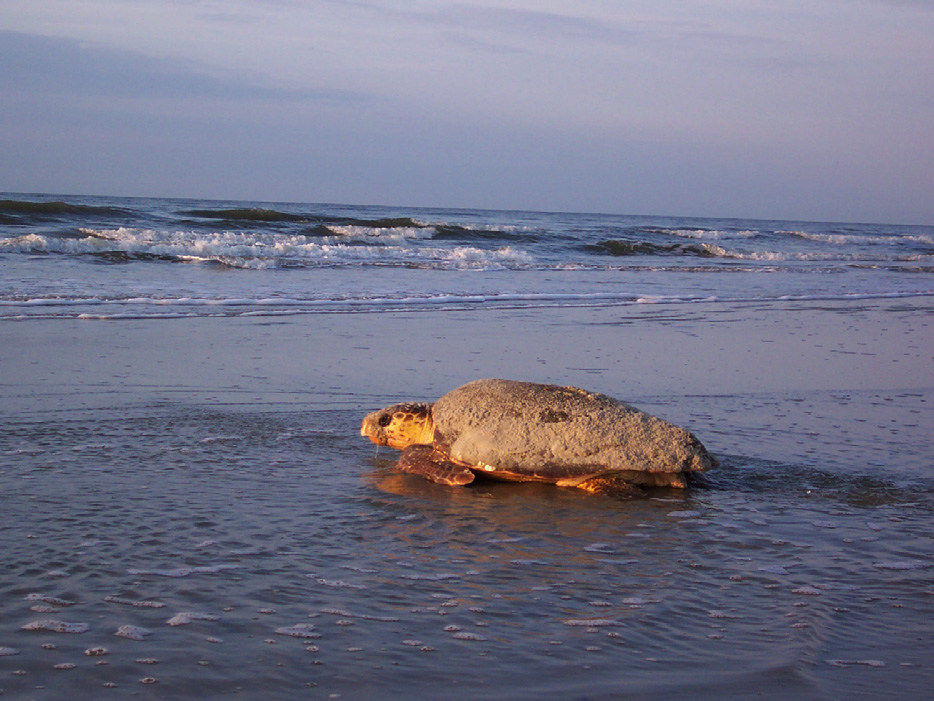
Loggerhead leaving the beach in the dawn on Wassaw Island, Georgia, USA.

In 2008, using GERUD, multiple paternal contributions were found in 19 of 23 clutches (82.6%), with an average of 3.00 (SE ± 0.23, Range: 1–6) males per clutch (COLONY: 19/23, 82.6%, 3.00 (SE ± 0.35, Range: 1–6)). In 2009, using GERUD, multiple paternal contributions were found in 15 of 21 clutches (71.4%), with an average of 2.62 (SE ± 0.38, Range: 1–6) males per clutch (COLONY: 18/21, 85.7%, 2.80 (SE ± 0.35, Range: 1–7)). In 2010, using GERUD, multiple paternal contributions were found in 18 of 28 clutches (64.3%) with an average of 2.21 males per clutch (COLONY: 19/28, 67.9%, 2.32 (SE ± 0.25, Range: 1–6)). When all 3 years are combined, using GERUD, multiple paternal contributions were found in 52 of 72 clutches (72.2%) with an average of 2.58 (SE ± 0.17, Range: 1–6) males per clutch, 95% CI: 2.24–2.93 (COLONY: 56/72, 77.8%, 2.72 (SE ± 0.18), Range: 1–7, 95% CI: 2.36–3.09) (Table 3, Fig. 1). Averaging the 3 years of data, 54 of the 72 clutches sampled had multiple fathers with an average of 2.65 (SE ± 0.13) males contributing to each clutch (Table 3).

**Table 3:** Descriptive table of multiple paternity by year, using GERUD, COLONY, and the average between the two programs. The second number is the average number of fathers per clutch and Standard Error.

Year	GERUD	COLONY	Average
2008	19/23 = 82.6%	19/23 = 82.6%	19/23 = 82.6%
	3.00 (±0.29)	3.00 (±0.35)	3.00 (±0.23)
2009	15/21 = 71.4%	18/21 = 85.7%	16.5/21 = 78.6%
	2.62 (±0.38)	2.80 (±0.35)	2.71 (±0.25)
2010	18/28 = 64.3%	19/28 = 67.9%	18.5/28 = 66.1%
	2.21 (±0.23)	2.32 (±0.25)	2.67 (±0.17)
Average	52/72 = 72.2%	56/72 = 77.8%	54/72 = 75%
	2.58 (±0.17)	2.72 (±0.18)	2.65 (±0.13)

### Contributing males per clutch

We estimated the number of males contributing to the Wassaw Island clutches over 3 years. Using COLONY, we estimated 195 individual male genotypes over all 3 years from the 72 clutches. The analysis produced using COLONY identified no half siblings.

Our conservative estimate (via GERUD) illustrated that over all 3 years, 20 clutches had 1 father, 17 clutches had 2 fathers, 21 clutches had 3 fathers, 7 clutches had 4 fathers, 3 clutches had 5 fathers, 2 clutches had 6 fathers, and 2 clutches had more than 6 fathers. Overall, 22 of 52 clutches (Table 3) with multiple fathers deviated significantly from the expected number of equal contributions among fathers. The upper range estimate (via COLONY) indicates 16 clutches had 1 father, 23 clutches had 2 fathers, 16 clutches had 3 fathers, 7 clutches had 4 fathers, 4 clutches had 5 fathers, 4 clutches had 6 fathers, and finally, 2 clutches had 7 fathers. Overall, 23 of the 56 (Table 3) clutches with multiple fathers deviated significantly from equality. GERUD cannot determine paternity accurately if there are more than 6 potential sires (Jones 2005), so there are two nests in our analysis that could only be analyzed by COLONY.

### Generalized linear model

**Figure 2:**
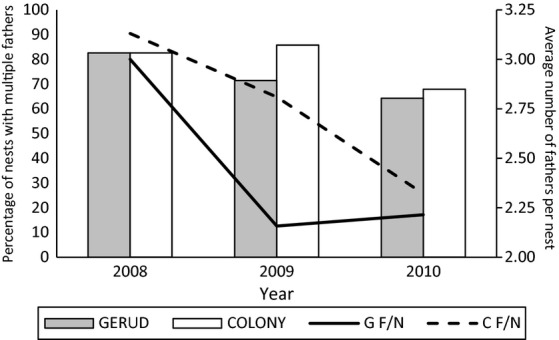
Graph showing the relationship between the two programs used and the 3 years analyzed. (*N*_2008_ = 23, *N*_2009_ = 21, *N*_2010_ = 28). The gray bars are GERUD by year, the white bars are COLONY by year, G F/N represents the number of fathers per clutch according to GERUD, and C F/N represents the number of fathers per clutch according to COLONY

**Figure 3:**
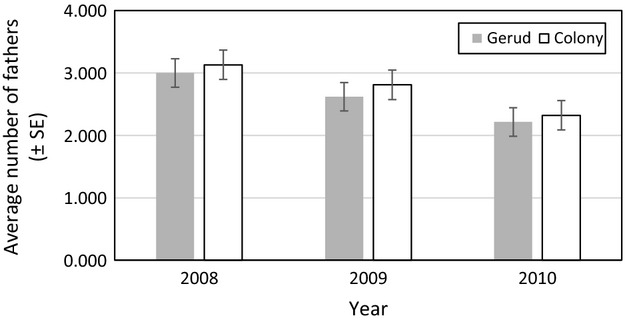
Average number of fathers by year using both estimator methods. There is a significant negative relationship between the number of fathers due to year (G: χ^2^ = 4.05, *P* = 0.0441; C: χ^2^ = 3.96, *P* = 0.0465).

Our last goal was to determine whether the number of fathers per clutch correlated with female size, the year sampled, Julian date, or hatching success. The conservative generalized linear model (Table 4B) showed that when all three variables (year, Julian nest date, and female size) were included, the variation in number of fathers per nest was not explained significantly by any independent variable (*P* = 0.130, 0.741–0.828, 0.205, respectively). However, when year and female size were analyzed without Julian nest date, both variables explained significant variation in the number of fathers per clutch (χ^2^_(1,71)_=4.05, *P* = 0.044 and χ^2^_(1,72)_ = 5.02, *P* = 0.025, respectively). The number of fathers per clutch decreased over the course of the study (χ^2^_(1,71)_ = 4.05, *P* = 0.044; χ^2^_(1,71)_ =3.96, *P* = 0.047, Fig. 2). As female size increased, the number of fathers increased (χ^2^_(1,71)_ = 5.02, *P* = 0.025; χ^2^_(1,71)_ = 4.63, *P* = 0.032, Fig. 3). The number of fathers per clutch did not explain variation in clutch hatching success (χ^2^_(1,71)_ = 1.93, *P* = 0.165). The less conservative (COLONY) generalized linear model (Table 4B) showed that when all three variables (year, Julian nest date, and female size) were included, the variation in the number of fathers per clutch was not explained significantly by any independent variable (*P* = 0.171, 0.661–0.798, 0.209, respectively). However, when year and female size were analyzed without Julian nest date, both variables explained significant variation in the number of fathers per clutch (χ^2^_(1,71)_ = 3.96, *P* = 0.047 and χ^2^_(1,71)_ = 4.63, *P* = 0.032, respectively). The number of fathers per clutch decreased over time (Fig. 2); as female size increased, the number of fathers per clutch increased (Fig. 3). The number of fathers per clutch does not explain variation in clutch hatching success (χ^2^_(1,71)_ = 0.97, *P* = 0.326).

**Table 4:** Generalized Linear Model for (A) GERUD, (B) COLONY distribution gamma. All variables have 1 degree of freedom, N = 72, chi-square values are first, followed by the *P*-value.

Parameter	Initial Model	Step 1	Step 2	Run Alone
(A)				
Year (2008–2010)	2.29, 0.1303	2.34, 0.1259	2.18, 0.1394	**4.05, 0.0441**
Straight Carapace Length (cm)	1.61, 0.2050	2.17, 0.1408	3.34, 0.0676	**5.02, 0.0251**
Julian Nest Date	0.09, 0.7656			
Hatching Success				1.93, 0.1645

(B)				
Year (2008–2010)	1.87, 0.1712	1.91, 0.1675	1.80, 0.1803	**3.96, 0.0465**
Straight Carapace Length (cm)	1.58, 0.2091	1.54, 0.2152	2.91, 0.0880	4.63, 0.315
Julian Nest Date	0.07, 0.7981			
Hatching Success				0.97, 0.3258

Significant at *P* less than 0.05 are in bold.

Finally, female size differed among years (*F*_(2,65)(0.05)_ = 4.77, *P* = 0.012), females in 2010 were significantly smaller. There was no difference in clutch size due to the number of fathers (G: χ^2^_(1, 71)_ = 0.16. *P* = 0.690; C: χ^2^_(1,71)_ = 0.24. *P* = 0.626).

## Discussion

This is the first study to investigate paternity and operational sex ratio in the Northwest Atlantic Ocean discrete population segment and the recovery plan's northern management (NMFS & USFWS, 2008) unit of loggerhead marine turtles. We report a frequency of 75% multiple paternity in a breeding population of loggerhead marine turtles; and a male/female by clutch ratio of 2.65/1. Further, our study is the first to report that, over 3 years of sampling, each male genotype was found only once. As no male sired more than one clutch throughout the 3 years, we suggest that this nesting population's mating system is polyandrous and not polygynous.

Current population models of the loggerhead assume that the adult male to female ratio is 1:1 (Conant et al. 2009). Our findings suggest that the operational sex ratio may be as high as 2.65 males per 1 female. The current conservative estimate for the number of reproductive females within the Northwest Atlantic Ocean is approximately 38,334 (Richards et al. 2011). If we assume that the population has a 1:1 sex ratio, then there should be 38,334 males. Extrapolating from this maximum, we can examine the likelihood that we would find 195 distinct males within this maximum population size: there is a 50% chance that we would have found 195 independent individual males over the course of the study. If instead, we use our ratio (2.65*38,334 = 101,585 males), there is an 81% chance that we could find 195 independent individual males (in a population *n*, these numbers represent the likelihood that none of the 195 are the same). This leads us to conclude the number of reproductively active males in the population has been considerably underestimated. If correct, one implication is that the Northern portion of the discrete population segment is not as vulnerable as current models suggest. If our high rate of multiple paternity is an indicator of a large number of adult males in the offshore waters, the effective population size may be stable or even growing.

Our results are consistent with population genetic data indicating high levels of male-mediated gene flow among the Northwest Atlantic nesting populations while female population structure exists due to nesting beach fidelity (Bowen 2005; Bowen et al. 2005). The high male to female by clutch ratio estimates at Wassaw would result from a large male population migrating along the coast and mating across nesting beach boundaries. Recently, a study tracked male movement of green turtles between nesting beaches during one breeding season suggesting this behavior is common among marine turtles (Wright et al. 2012a,b). This mating behavior was interpreted to be an adaptive response to climate change. The movement of males between rookeries appeared to raise the sex ratio. Further, Watanabe et al. (2011) reported that genetic diversities in microsatellite loci for nesting females on a small nesting beach were highly similar to larger nesting beaches in Japan. They suggested that interbreeding must occur during migration between females and males from different populations to maintain nuclear genetic diversity of the whole Japanese population (the North Pacific Ocean). The movements of the males that contribute to the loggerhead clutches on Wassaw Island have not been tracked and this could be an avenue for future study.

**Figure 4:**
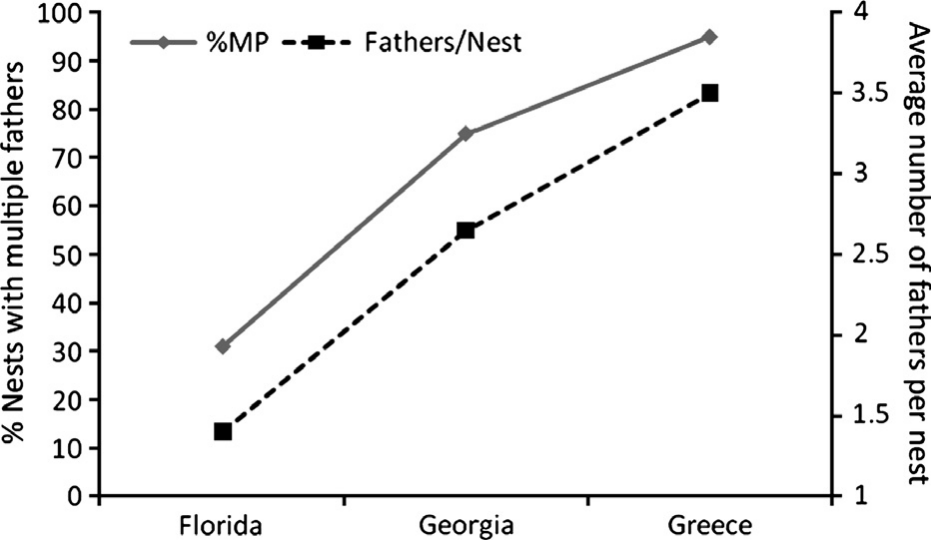
Relationship between nesting location, percentage of clutches sired by multiple males, and the average number of fathers per clutch by location. Adapted using data from Moore and Ball 2002 and Zbinden et al. 2007.

Although there are few data concerning the number of reproducing males, a relationship between the number of nesting females in a population and the frequency of multiple paternity has been suggested (Jensen et al. 2006). When olive ridley turtles, *Lepidochelys olivacea,* nest singularly, 30% of clutches have multiple paternal contributions, but within an olive ridley arribada (mass nesting) beach, the frequency rose significantly to 92% (Jensen et al. 2006). This difference is attributed to the abundance of individuals in the mating system, suggesting that more males attend to larger numbers of receptive females (Jensen et al. 2006). Following this assessment, larger nesting beaches (more mating females) might be predicted to have a higher percentage of clutches with multiple paternity. This pattern may not hold true for loggerheads where two large nesting populations in Florida and Greece were found to have 31% and 95% of clutches with multiple paternal contribution, respectively (Moore and Ball 2002; Zbinden et al. 2007). Our estimates for the small rookery at Wassaw suggest that the rate of multiple paternity is nearly double than that of the large south Florida rookery (Moore and Ball 2002), but less than estimates from the Mediterranean Sea discrete population segment in Zakynthos, Greece (31 < 75 < 95%, Fig. 4) (Zbinden et al. 2007). The average minimum number of fathers per clutch show a similar pattern (1.4 < 2.65 < 3.5, Fig. 4) (Moore and Ball 2002; Zbinden et al. 2007). This raises the question as to whether the density of females, rather than purely large nesting numbers, might explain differences in the number of clutches with multiple fathers in a nesting population. It is also possible that expected sex ratios of hatchlings affect how many males return to their natal beaches as adults. Loggerhead clutches in higher latitudes (North Carolina–Northern Florida) are currently expected to produce between 15 and 45% males (Mrosovsky 1984; Bell, 2003; Hawkes et al. 2007; LeBlanc et al. 2012), whereas clutches in Southern Florida are currently expected to produce upwards of 90% female hatchlings (Mrosovsky and Provancha 1992; Witt et al. 2009). These data suggest that there would be a higher proportion of males produced on Wassaw Island, GA, than from Florida nesting beaches, which in turn would increase the proportion of males contributing to Wassaw's nests.

Biogeographical factors such as small area of breeding grounds could positively influence the occurrence of multiple paternity (Zbinden et al. 2007). The ocean floor off Wassaw Island drops sharply in a feature known as the Georgia Bight. If the Bight limits the size of the area in which loggerheads congregate off Wassaw, a dense concentration of turtles may result. This hypothesis could be tested by sampling from a similarly small nesting beach in Florida to see whether there arises a difference in reproductive turtle concentrations. Even if biogeographical effects were determined to be a significant variable in multiple paternity, such a finding would not rule out other variables arising from genetic differences among the subpopulations within the Northwest Atlantic Ocean discrete population segment.

Recently, Shamblin et al. (2011) discussed the “northern management unit” as genetically distinct from the “southern management unit,” potentially suggesting that the northern nesting beaches were colonized earlier by *C. caretta*. Our data may indicate that mating behavior differs between north and south management units. However, the abundance of nests in the southern management unit and the large geographic range of nesting make it challenging to collect a sample size large enough to explain this difference with statistical significance. Studies with more robust behavioral analysis coupled with molecular methods in the southern management unit could test this notion of behavioral differences. Once researchers understand whether or how mating systems vary from beach to beach or among subpopulations, then managers can begin to provide species-specific and population-specific plans for conservation.

Our study found that the number of fathers increased as female size increased. Previous studies have shown that nesting females continue to grow, suggesting that older females are typically larger (Cason 2009; Casale et al. 2011). Older females have had another year in which to store sperm; increased capacity for sperm storage may lead to increased multiple paternity. However, recent captive studies (low sample size) suggest that females do not store sperm for longer than 1 year (Sakaoka et al. 2013). Further, males may favor larger females because larger females have a higher capacity to hold eggs (Van Buskirk and Crowder 1994); however, recent studies of green sea turtles suggest that this may not be the case (Wright et al. 2013). The size of the females in our study varied significantly by year. Specifically, females in 2010 were smaller, suggesting that these turtles could be younger than turtles from the previous 2 years. Hence, the number of fathers may also reflect the mating behavior of naïve nesters.

We found no difference in the number of fathers per clutch due to nesting date. This is consistent with sperm mixing at the beginning of the season (Pearse et al. 2002; Uller and Olsson 2008). Females store sperm in their oviduct after mating (Uller and Olsson 2008). If sperm of successive males were stored in sequence, we might see a skew in the number of fathers per clutch over the course of the season. However, as there was no pattern of multiple fathers or single father's contribution, sperm must have mixed. Recent studies investigating successive clutches of individual female leatherback and hawksbill turtles support this claim of mixing rather than stratification (Stewart and Dutton 2011; Phillips et al. 2013). However, the order of sequential matings may also affect paternity (Sakaoka et al. 2011).

Multiple paternity may be favored if it increases the variability and viability of the offspring (Lee and Hays 2004; Uller and Olsson 2008; Wright et al. 2013), but these consequences are not necessarily related. A simple way to assess viability is to examine hatching success, defined here as the rate of hatchlings escaping the nest. There was no relationship between hatching success and the number of fathers per clutch. This could suggest that more fathers add to the variability but not to the viability of hatchlings. We propose that with more fathers, there would be more variability, and so alleles related to viability would also be variable. However, our study did not sample dead hatchlings in the nest. It is possible that there is a bias due to our sampling, only hatchlings that made it out of the nest.

The data presented here have the potential to challenge current population models as well as long-held behavioral models. Our findings suggest that the 1:1 male to female ratio in the Northwest Atlantic Ocean should be reevaluated or that our behavioral models of turtles mating just off the nesting beach and the duration of female receptivity should be revisited. Current understandings of hatchling sex ratios raise concerns about the number of males at hatching. Perhaps, the more critical concerns should be to determine just how many females are surviving to maturity, as well as how male-mediated gene flow is actively affecting the operational sex ratio of our nesting beaches. This study is limited to a single small nesting beach, yet the results were unexpected and relate directly to population estimates and metrics for population recovery. For these reasons, we suggest it is important that paternal studies that estimate numbers of males contributing to nesting assemblages be expanded to other nesting beaches. This should include both small and larger nesting sites to the south to reevaluate our current understanding of paternal contributions.
